# Universal Safety Planning for Suicide Prevention: CODE RED Initial Feasibility and Acceptability

**DOI:** 10.3390/ijerph21121704

**Published:** 2024-12-21

**Authors:** Julie Cerel, Martina Fruhbauerova, Alice Edwards, Leah Murphy, Elizabeth Salt, Beck Whipple, Patti M. Clark, John Ackerman

**Affiliations:** 1 College of Social Work, University of Kentucky, Lexington, KY 40506, USA; alice.edwards@uky.edu (A.E.); leah.murphy@uky.edu (L.M.); 2 Department of Psychology, University of Kentucky, Lexington, KY 40506, USA; anitram@uky.edu; 3 College of Nursing, University of Kentucky, Lexington, KY 40506, USA; elizabeth.salt@uky.edu; 4 Kentucky Department for Behavioral Health, Developmental and Intellectual Disabilities, Frankfort, KY 40621, USA; beck.whipple@ky.gov (B.W.); patti.clark@ky.gov (P.M.C.); 5 Nationwide Children’s Hospital, Columbus, OH 43205, USA; john.ackerman@nationwidechildrens.org

**Keywords:** suicide, prevention, school, universal

## Abstract

Suicide rates have increased in the US over the last decades. Schools often deliver suicide prevention trainings and there is growing evidence that these trainings in schools are effective. The current study examined a new upstream approach, CODE RED in which trainees complete their own safety plan prior to a mental health emergency. Participants were adult school personnel (*n* = 201) who completed CODE RED trainings and were surveyed using three validated 4-item measures: acceptability of intervention measure (AIM), intervention appropriateness measure (IAM), and feasibility of intervention measure (FIM). Open-ended questions were analyzed using a thematic approach. Of 201 participants who completed the survey, acceptability (18.0), appropriateness (17.9) and feasibility (18.0) were high as assessed by standardized implementation measures (each out of 20). Open-ended responses further indicated a great deal of satisfaction with the training. As a first step in determining acceptability and feasibility, CODE RED was found to be highly acceptable to adult school employees who found it applicable, appealing as an intervention, and easy to use. Most staff are hopeful that it will be useful with youth as well. It will be important to determine how this activity is used by youth and if it can be used in mental health crises to decrease symptoms.

## 1. Introduction

There are over 48,000 suicides each year in the U.S., and suicide is a leading cause of death among youth and young adults [1]. Despite extensive efforts to address mental health issues and prevent suicide, the suicide rate has continued to climb over the last two decades particularly in youth, suggesting current approaches are likely limited by reach, effectiveness, and sustainability [2]. Data from the Youth Risk Behavior Survey (2021) indicate that 22.2% of students reported seriously considering suicide in the past year. Additionally, 1 in 10 (10.2%) high school students indicated they had made a suicide attempt within the past year and almost 2.9% received medical attention for a suicide attempt (CDC, 2021).

Recent efforts to address suicide prevention include the development of a new three-digit suicide crisis line 988, which was unveiled in 2022, as well as required suicide prevention training in schools across 17 states. However, awareness of 988 by those who would be most likely to experience suicidal thoughts or intervene to prevent suicide is lacking [3], and state requirements and guidelines are often vague or underdefined in state laws [4]. In addition, the effectiveness of both crisis hotlines and training relies on the availability and willingness of an external individual to intervene, or on the suicidal person’s ability to reach out for help during a crisis. Since individuals in crisis may struggle to seek help or find that available support does not meet their immediate needs, there is an urgent need to develop innovative, proactive solutions.

Empowering individuals to prevent injury, especially youth, is a well-established public health approach. Emergency preparedness and public health initiatives recognize the utility and advantages of planning [5]. For example, schools implement and practice a variety of safety drills (i.e., weather, fire, lockdown) and fire drills (i.e., stop, drop, and roll) with students so that should an emergency occur, students will be prepared and know how to respond. Similarly, public health campaigns encourage individuals to create plans that will increase the likelihood that they engage in healthy or protective behaviors (e.g., fire safety plans). Critically, both emergency preparedness and public health efforts focus on motivating individuals to create a plan and rehearse their actions before an event occurs.

Like emergency preparedness and public health efforts, mental health safety planning and crisis planning interventions have been developed. However, they are typically only implemented during or after a crisis. For example, the Safety Planning Intervention (SPI) [6], a widely used and validated suicide intervention, (see [7]), involves recognizing personal warning signs, identifying effective coping mechanisms, creating a list of trusted adults, and implementing measures to restrict access to lethal means. However, SPI is often initiated after an individual has already experienced suicidal thoughts or behaviors that precipitated acute intervention (e.g., in an emergency department, on an inpatient psychiatric unit). As such, it is a reactive approach. This is problematic given our limited ability to predict suicide accurately [8].

In fact, research consistently shows that a considerable number of suicides move from thought to action without prior planning [9,10,11,12], and some individuals attempt suicide without experiencing suicidal thoughts beforehand [13]. Moreover, the intensity and onset of suicidal ideation can vary significantly over time [14]. Additionally, most suicides occur among individuals categorized as having low or medium risk when assessed by a provider [15]. These findings suggest that a significant portion of suicide deaths or attempts occur in an unplanned manner, rendering reactive intervention efforts ineffective due to the spontaneous nature of the act. Thus, it would be prudent to establish universal suicide or mental health interventions, akin to the widely taught “stop, drop, and roll” fire safety technique, that are implemented and practiced proactively, universally, and prior to the onset of a mental health or suicidal crisis.

Equipping people with skills on how to respond, when necessary, even in the absence of an immediate mental health crisis, can be invaluable. This proactive approach may be particularly crucial in schools, where rates of anxiety, depression, and suicide risk have surged among children and adolescents [16,17,18,19,20,21]. To address gaps in proactive mental health or suicide crisis prevention, a universal suicide prevention program known as CODE RED was developed. The purpose of this study was to evaluate the implementation of CODE RED with adults in a school context. This approach involved training all adults in the school to create their own safety plans before extending the training to youth. Thus, this evaluation examined the feasibility and acceptability of the intervention for adults.

## 2. Materials and Methods

Using a cross-sectional design, 201 adult participants completed the CODE RED implementation evaluation following a one-hour training in which they completed their own personal CODE RED safety plan during the 2022–2023 school year. Participants came from across the Commonwealth of Kentucky and were trained at 12 sessions throughout the state. Training was provided to school staff across all levels: elementary, middle, and high school, as well as to support personnel including bus drivers, clerical staff, and cafeteria staff. The goal was for these adults to create their own CODE RED plans to ensure that they have a plan for their “worst day.” This initiative was part of the Kentucky Garrett Lee Smith Youth Suicide Prevention Grant. This study gathered preliminary data on the acceptability, appropriateness, and feasibility of this program among adults in school communities. This initial evaluation aimed to assess whether the program was considered worthwhile by the adults and to avoid the need for extensive school approval and consent that would be required for youth evaluations. This marks the first collection of data from participants who took part in a CODE RED training.

### 2.1. Intervention

**Figure 1:**
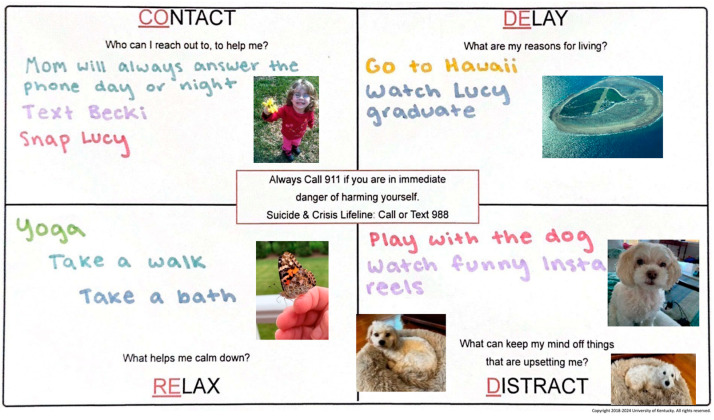
CODE RED example. COntact, RElax, DElay, Distraxct.

Developed by the first author, CODE RED is a new universal safety planning intervention designed to collaboratively create a plan with individuals before they experience what is referred to as their “worst day”. CODE RED stands for “COntact”, “DElay Decisions”, “RElax”, and “Distract” and encourages individuals to create a safety plan by identifying trusted people to contact, delaying risky decision-making, and finding activities to relax and distract from thoughts of suicide. The program can be delivered to both adolescents and adults in a one-hour interactive group training session. During the training, individuals complete their own safety plans while the group leader provides instructions and shares their personal plan as an example. Completion of CODE RED training results in a physical or electronic plan with all the necessary information for future use (see Figure 1). Individual plans are completely personalized and may include written notes, personal photos, drawings/art, or images from magazines or the internet.

This work was inspired in part by 4 Mental Health’s Staying Safe (https://www.stayingsafe.net/). It was developed and piloted in partnership with Nationwide Children’s Hospital’s Boys and Girls Clubs of Central Ohio in 2020, and with School Nurses in Kentucky in the BARN to Nursing State project in 2022. However, neither of these pilot studies collected data about CODE RED.

Each plan starts out with four blank quadrants, with the 988 Crisis and Suicide Prevention Lifeline number and any relevant local crisis numbers prominently displayed in the center of the document (see Figure 1). A trained CODE RED instructor leads the participants through the exercise.

In the “Contact” quadrant, individuals are encouraged to list the phone numbers of mental health professionals they are currently seeing (if applicable), responsible adults who can respond in a crisis (such as family members or other adults in the community, like teachers or the school nurses), and trusted peers. Due to the importance of social support and not feeling alone in a suicidal crisis, the contact quadrant helps people generate those social supports who could be there for them in a crisis.

The “Delay Decisions” quadrant prompts individuals to list “things you can think about that give you motivation and hope” including future plans and personal reasons for living.

In the “Relax” quadrant, participants identify calming or self-regulating activities. These might include formal techniques like deep breathing or progressive muscle relaxation, as well as more personal activities like taking a warm bath, listening to music, or journaling.

Finally, the “Distract” quadrant focuses on “anything you can do to keep your mind off the situation” These activities aim to help divert the person’s mind from their immediate distress for a few minutes or hours. This might involve engaging in hobbies, watching a movie, or exercising until the acute situation resolves or professional help can be obtained (see Figure 1).

CODE RED instructors take steps to ensure that the coping strategies listed do not increase risk, avoiding options like using alcohol and other substances, or engaging in risky activities such as visiting a gun range or hunting.

### 2.2. Measures

The survey included basic demographics and three widely used implementation science measures: the Acceptability of Intervention Measure (AIM), Intervention Appropriateness Measure (IAM), and Feasibility of Intervention Measure (FIM; [22]. Each of these measures includes four items which are scored from 1 (completely disagree) to 5 (completely agree). Thus, the total score for each measure can range from 4–20, with a composite score across all measures ranging from 12–60 with higher total scores indicating greater agreement. This brief measure takes less than five minutes to complete. Internal consistency for this measure in the sample was high (McDonald’s ω = 0.98)

Demographic data collected encompassed information regarding the individual’s role within the school and the school’s educational level (elementary, middle, high school, or mixed). The educational level was open to participants to check all options that apply. However, due to constraints related to time, study type, and privacy, additional demographic details such as gender and age were not collected.

### 2.3. Procedures

All participants underwent a one-hour CODE RED training led by grant personnel in which they made their own CODE RED plan. Some of the plans were online and some were on paper. Participants were asked to bring pictures or cut images out of magazines. After the conclusion of the CODE RED training, participants completed a brief voluntary evaluation. This anonymous pencil and paper survey was approved by the university IRB.

### 2.4. Analytic Approach

The primary feasibility and acceptability outcomes, which include AIM, IAM, and FIM, were computed by aggregating the responses from the individual questions. To provide a comprehensive overview of the data, means, standard deviations, and ranges are reported for the measurement scales individually. A composite score across all three measures, ranging from 12–60 was also calculated. Themes were generated using a grounded theory approach in which themes were generated from the data itself with no a priori hypotheses. Themes were generated and coded as present or absent by two authors who agreed with all coding. Coding was done in Microsoft Excel. When there was disagreement on codes, authors met for a consensus conference and brought in a third author when consensus could not be reached.

## 3. Results

The breakdown of participants’ employment across educational levels was as follows: 40.3% High School (*n* = 81), 18.4% Elementary (*n* = 37), 9.5% Middle School (*n* = 19), 6.5% two or more school settings (*n* = 13), and 25.4% other, roles extended beyond specific school levels to include district-wide professions.

Teachers constituted the largest group among school roles (*n* = 80, 38.8%). Other roles included prevention specialist or family resource coordinator (*n* = 25, 12.4%), bus drivers (*n* = 15, 7.5%), administrators (*n* = 11, 5.5%), cafeteria workers (*n* = 11; 5.5%), counselors (*n* = 8, 4.0%), custodial staff (*n* = 8, 4.0%), instructional aid or paraprofessional (*n* = 5, 2.5%), and other (*n* = 38, 18.9%). Other categories included single respondents from a wide range of school personnel including practicum students, nurses, speech pathologists, daycare workers, and youth services coordinators.

**Figure 2:**
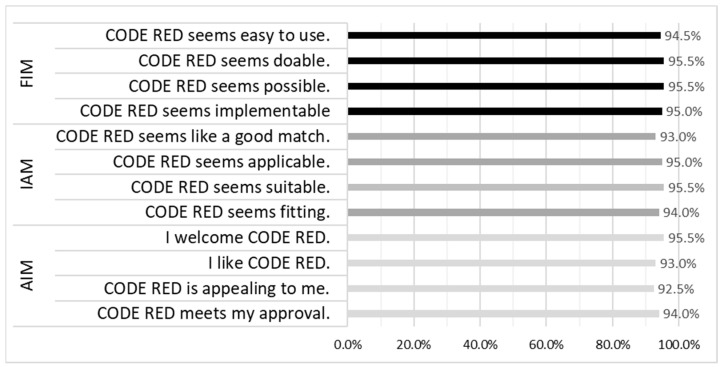
Accessibility, Appropriateness and Feasibility of CODE RED (Note: Acceptability of Intervention Measure (AIM), Intervention Appropriateness Measure (IAM), and Feasibility of Intervention Measure (FIM)). Percent who agree or strongly agree.

The implementation measures, AIM, IAM, and FIM, were employed to gauge respondents’ perspectives on the acceptability, appropriateness, and feasibility of introducing CODE RED into their schools. The means, along with their corresponding standard deviations and ranges, for these measures were as follows: AIM averaged 17.96 (SD = 2.5; range = 4–20); IAM averaged 17.95 (SD = 2.6; range = 4–20); and FIM averaged 18.01 (SD = 2.5; range = 4–20). For the total measure, the mean was 53.92 (SD = 7.3, range = 12–60). Figure 2 shows the proportions of respondents who either agreed or completely agreed with these implementation measures, shedding light on their positive attitudes towards the adoption of CODE RED in their respective schools.

Each evaluation included an open-ended response for participants to indicate if they had any further feedback about the training. Of the 201 people who participated in the evaluation, 42 responses were received. Of these, 29 described the training as positive. This was best exemplified by comments such as: “So much better than a boring video that no one pays attention to. Helpful to participants on a personal level and provides a common language to use throughout the school building” and “A very universal and effective suicide prevention training. One of my favorite so far. It was nice to focus on the individual safety vs. suicide prevention as a whole”.

Ten people commented on how the activity would be good for teens in their schools with statements such as “I feel like this training will help me talk easier with the young children in my own family, as well as the young people in my school” and “Easy to use! Most students would connect well with this activity”. Four people commented on the flexibility of doing a plan by hand or on a computer and taking a picture. These comments included: “on the computer or on a hard copy—either way it is easy to complete” and ”I like them doing it—having a visual and taking a photo on their phone”.

Finally, two people expressed concern about the training but both were not about the training itself but about the need to have appropriate support in place when CODE RED is offered to students. These statements were “I am concerned about the ability to provide the necessary immediate services to students who are triggered by the training. It requires some intensive personal work and is emotionally heavy material. Given the size of counseling caseloads in the high school setting, lack of other school level support services, and the fact that we will be helping to deliver the training to students”, and “I am concerned about having enough support to manage individual student needs that come up during this process”.

## 4. Discussion

Data from this study indicate that CODE RED has a very promising level of overall acceptability, appropriateness, and feasibility when administered to adult school personnel which is an important, albeit preliminary, step. Participant feedback supported the attributes of the intervention that addressed significant gaps in prior training. Participants expressed satisfaction, highlighting its potential effectiveness, individualized approach, and engaging nature, stating CODE RED had a significant utility in addressing this health concern with the youth they interact with. Further, some participants expressed that this universal safety planning approach would facilitate discussions about mental health with children, making it easier to use and for children to relate to. Participants also expressed concerns about potential crises that children might encounter during the training, noting the “heavy nature of the content”. This concern is often raised by school staff in the context of suicide prevention and screening efforts, yet there is no evidence to suggest that discussing the topic of suicide in a responsible manner with trusted adults would precipitate a suicidal crisis in individuals [23]. These comments suggest additional training is needed to improve the understanding of universal preventative interventions for suicide and how stigma about suicide gets in the way of prevention efforts.

In fact, a unique and potentially appealing quality of employing CODE RED is suicide prevention implemented as a universal public health approach, which may confer several advantages to this preventative intervention. Specifically, the idea that safety planning isn’t exclusively for those currently in crisis or at imminent risk of a crisis, but beneficial for everyone, helps to diminish the stigma surrounding the anticipation of future mental health issues. By not explicitly using the term “suicide prevention”, CODE RED de-emphasizes a potentially stress-inducing topic among some educators—suicide prevention trainings. The universal aspect of this approach normalizes preparation for anyone and in turn, decreases the stigma that exists across society in preparing for a mental health crisis. Even people who do not anticipate being suicidal can think about how they might have a worst day. While many suicide-specific interventions are typically utilized after a crisis has occurred, CODE RED assumes that everyone is at risk of a mental health crisis at some point in their lives. By proactively anticipating, preparing for, and coping with the possibility of a “worst day”, individuals are better equipped to navigate such situations should they arise. In other words, creating and implementing a plan before experiencing a crisis, as opposed to after, may increase the likelihood of devising more effective strategies and being more inclined to use them. Consequently, CODE RED may have the potential to prevent unnecessary psychiatric hospitalizations, suicide attempts, or even suicides.

Another key strength of this universal safety planning approach is that it is designed for ease of dissemination. CODE RED can be effectively administered by laypeople, making it accessible for widespread implementation in various settings. This simplicity allows for a broader reach, enabling more individuals to benefit from the program without the need for specialized training. As a result, communities can equip more people with the tools they need to support themselves and others during a crisis, ultimately enhancing the overall impact of suicide prevention efforts.

Areas for future research involve determining whether adults implement their CODE RED plans and whether this influences their willingness to intervene with others in distress. Further research should also explore if personal usage of these plans is related to reductions in suicidal ideation and behaviors, and symptoms of distress such as depression and anxiety. These studies need to be done with diverse populations in different settings (i.e., rural and urban, school and community) across more than one state. Future studies should also include more extensive demographic measures such as age, sex, and ethnicity to further evaluate the generalizability and applicability of the data obtained in this study. As there is no research on normalizing this preparation, this will need to be the topic of future study. Another strength of CODE RED is that it is a prevention approach not just for individuals who have already experienced a mental health crisis or attempted suicide but for all.

## 5. Conclusions

CODE RED is a unique preventive public health crisis intervention that empowers individuals to proactively prevent suicide, which is a fundamentally different approach from those typically implemented. Critically, CODE RED was designed not just for individuals who have already experienced a mental health crisis or attempted suicide, but for everyone. Rather than focusing on those deemed at risk of suicide, CODE RED presumes anyone could become suicidal and was therefore designed to be as accessible and easy to implement as possible. Data obtained in this study suggest CODE RED is successful in this regard. Specifically, results from this implementation evaluation provide support for the acceptability, appropriateness, and feasibility of administering CODE RED to adult school personnel. This data is consistent with the intent of CODE RED—to simplify the empirically supported components of other suicide-specific interventions into an easily remembered and accessible format prior to the onset of a crisis. This approach ensures that individuals can quickly recall and implement their safety plans during their worst moments. By focusing on simplicity, accessibility, and planning, CODE RED becomes a practical tool for suicide prevention, empowering individuals to have a plan in place so that they can take immediate action when they need it most.

## Data Availability

Data are available by contacting the first author.
